# Strategies for the Identification and Prevention of Survey Fraud: Data Analysis of a Web-Based Survey

**DOI:** 10.2196/30730

**Published:** 2021-07-16

**Authors:** Mandi Pratt-Chapman, Jenna Moses, Hannah Arem

**Affiliations:** 1 GW Cancer Center The George Washington University Washington, DC United States; 2 Milken Institute School of Public Health The George Washington University Washington, DC United States; 3 Healthcare Delivery Research Medstar Health Research Institute Washington, DC United States

**Keywords:** cancer survivors, pandemic, COVID-19, fraudulent responses, survey, research methods, cancer patients, fraud, CAPTCHA, data integrity, online surveys

## Abstract

**Background:**

To assess the impact of COVID-19 on cancer survivors, we fielded a survey promoted via email and social media in winter 2020. Examination of the data showed suspicious patterns that warranted serious review.

**Objective:**

The aim of this paper is to review the methods used to identify and prevent fraudulent survey responses.

**Methods:**

As precautions, we included a Completely Automated Public Turing test to tell Computers and Humans Apart (CAPTCHA), a hidden question, and instructions for respondents to type a specific word. To identify likely fraudulent data, we defined a priori indicators that warranted elimination or suspicion. If a survey contained two or more suspicious indicators, the survey was eliminated. We examined differences between the retained and eliminated data sets.

**Results:**

Of the total responses (N=1977), nearly three-fourths (n=1408) were dropped and one-fourth (n=569) were retained after data quality checking. Comparisons of the two data sets showed statistically significant differences across almost all demographic characteristics.

**Conclusions:**

Numerous precautions beyond the inclusion of a CAPTCHA are needed when fielding web-based surveys, particularly if a financial incentive is offered.

## Introduction

The COVID-19 pandemic resulted in significant delays to health care administration. To assess the impact of the pandemic on cancer survivors in the United States, the study team fielded a survey in the winter of 2020. The survey was promoted via email and, briefly, via social media. The volume of results in a short time period suggested that the data should be reviewed for fraudulent responses.

Social media can be an efficient way to disseminate web-based surveys [1-5]. According to the Pew Research Center, in 2021, 72% of adults in the United States were estimated to use at least one form of social media [6]. However, ensuring data integrity of studies when using social media remains a challenge. This study describes the data integrity methods used to identify fraudulent and suspicious data in a web-based survey that was briefly open to the public via social media.

## Methods

### Participant Sample

We recruited cancer survivors primarily via an email request sent to physician liaisons and cancer registrars at institutions accredited by the Commission on Cancer (CoC). The study invitation, which came directly from the CoC, asked recipients to forward the invitation to their cancer center survivorship coordinator, who in turn was asked to forward the invitation to patients. Emails were sent on October 13, 2020, followed by two reminders, each 1 week apart. In addition, the study team disseminated the survey to community partners on October 8, 2020; posted on the Association of Community Cancer Centers eXchange and Association of Oncology Social Work listservs; and included the survey link in a George Washington University newsletter to health care professionals.

### Incentives

Participants were asked to complete a 20-minute survey and were told they would receive a US $25 gift card to thank them for their time.

### Precautions

To dissuade bots, we included a Completely Automated Public Turing test to tell Computers and Humans Apart (CAPTCHA), a question asking how the participant heard about the survey, time stamps, open-ended questions, and pairs of items that could be compared for consistency. After receiving over 1000 responses in the first 3 days after opening the survey, we examined the data and identified suspicious patterns. We then removed all links from social media and added additional precautions based on extant literature about optimizing valid responses for public-access surveys [7-9]: including a hidden item that could only be detected by bots, requiring participants to retype a word, and requiring participants to confirm their understanding that fraudulent responses would not be compensated.

### Measures

Our survey questions included demographics and health history: age, sex, and gender identity; sexual orientation; race/ethnicity; marital status; household size; education; income; age at diagnosis; cancer stage; cancer type; employment status; and insurance type. We also included questions related to COVID-19 and patient-reported outcomes.

### Data Cleaning

Data were exported from Research Electronic Data Capture (REDCap) and analyzed in SAS 9.4 (SAS Institute). As of Thursday, December 3, 2020, we had received 1977 responses. We thus developed criteria to identify suspicious and fraudulent data.

We began by eliminating those who were ineligible: respondents who were living outside of the United States, had stage 0 cancer, had no cancer diagnosis (n=83), or reported that they had only nonmelanoma skin cancer (n=46) [10]. We then eliminated respondents who were missing data on ≥35% of survey questions (n=149). Next, we excluded respondents who reported contradictory responses, including discordant gender (eg, both cisgender male and cisgender female status) (n=12) and discordant sex assigned at birth with anatomical site of cancer (eg, cisgender male with uterine cancer) (n=37).

**Figure 1:**
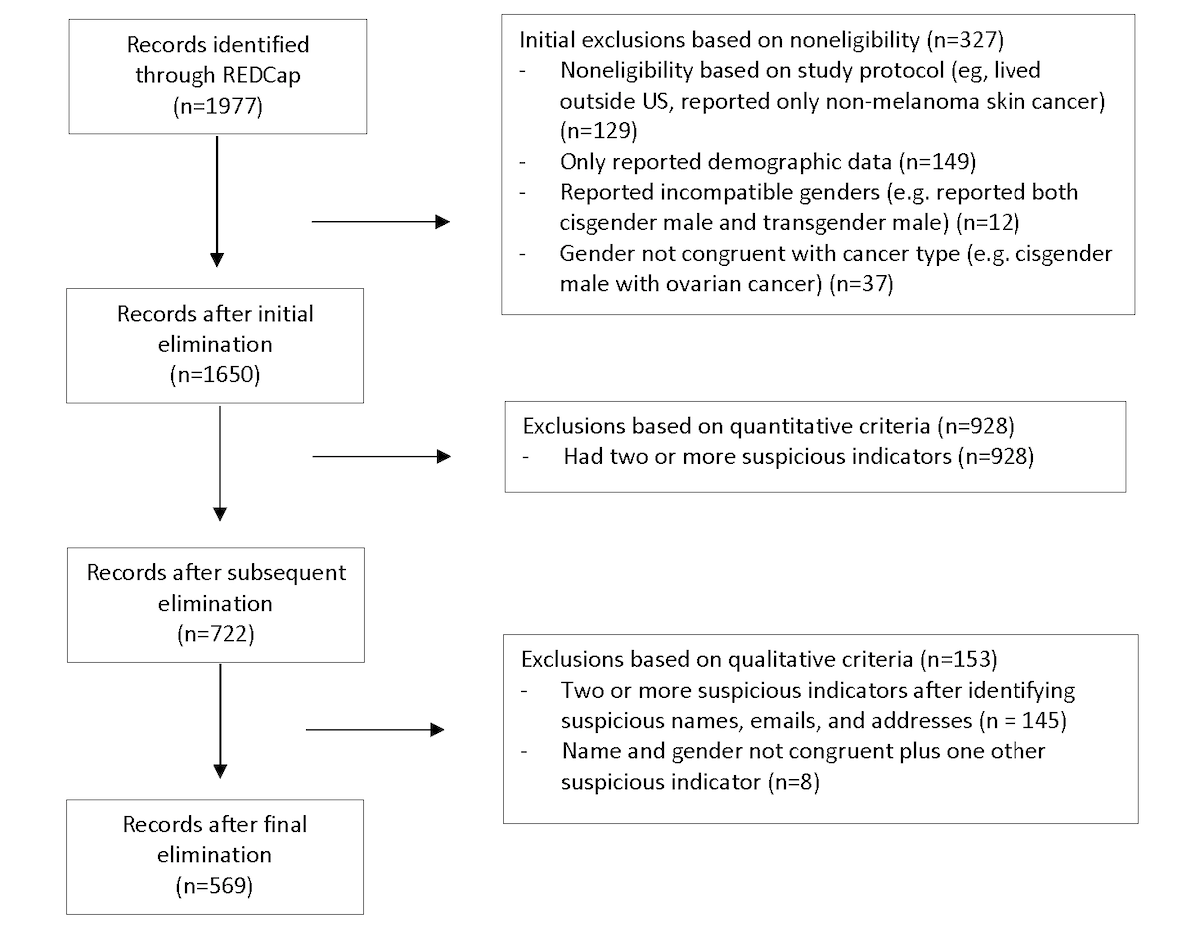
Flow chart of survey response elimination. REDCap: Research Electronic Data Capture.

We analyzed irregularities in the remaining data (n=1650) and eliminated responses that contained two or more suspicious indicators (Table 1). Criteria for a suspicious indicator included differences between reported and calculated age or reported and calculated time between treatment and diagnosis; report of a type of cancer that is very rare for the respondent’s age group; incongruent patterns of hearing about the survey relative to distribution dates; suspicious open-text responses (including fake addresses); repeat email addresses; and unusual time stamps. Table 1 presents a summary of the types of fraudulent and suspicious responses, and Figure 1 shows the elimination sequence.

We sent emails to all respondents excluded from the final data set to alert them that their responses had not passed a quality check, and we welcomed them to reach out to the study team with any questions. We received only 1 response, which said: “Why.” We also emailed all of the respondents who were retained in the data set and instructed them on how to claim their incentive. We received 1 response from a person who did not recall participating in the study. As additional quality control, we reviewed a subset of data for respondents who indicated hearing about the survey from a specific community partner. Of the 35 respondents who indicated hearing about the survey from this partner, we excluded 30. Upon member checking, all 5 participants retained in the data set were confirmed as clients of the community partner, and only 1 of the excluded respondents was a legitimate client.

**Table 1:** Types of fraudulent or suspicious data identified in eliminated survey responses (n=1081).^a^

Description	Value, n (%)
Year of birth is reported as 2020, or reported age and age calculated from reported date of birth are different by more than 1 year	250 (17.8)
Reported age is <40 years and cancer type is rare for those aged <40 years	283 (20.1)
Respondents indicate a survey source prior to dissemination of the survey from that source	820 (58.2)
Open-ended comments focus on information technology rather than answering the question asked	56 (4)
Open-ended telehealth comments are duplicates	34 (2.4)
Final open-ended suggestion responses are duplicates	107 (7.6)
Email addresses are duplicates	20 (1.4)
Time since diagnosis is <2 years, but time since treatment is 2-5 years	11 (0.8)
Time since diagnosis is ≤5 years, but time since treatment is >5 years	57 (4)
Suspicious survey time (at least 10 surveys completed in succession within 5 minutes of each other or completed between midnight and 4 AM EST)	986 (70)
Email/address is suspicious (for email: at least 10 random numbers or letters in a row, or strange punctuation or capitalization; for address: incomplete address, address of a business, address is not real, address includes quotation marks, or pattern of strange capitalization or spacing)	166 (11.8)
Name/suffix is suspicious (first and last name flipped, part of last name in first name field or vice versa, male suffix and female name, random letters or numbers in suffix field)	78 (5.5)

^a^Individuals could be counted in as many indicators as their responses suggested; thus, the n values do not add up to the total of excluded data.

### Data Analysis

After eliminating responses deemed as fraudulent, we used means and frequencies to create a demographics table comparing respondents who were included with those who were excluded. We used chi-square or Fisher exact tests to examine differences between groups.

### Ethical Review

This study was deemed exempt by the George Washington University Institutional Review Board (IRB) (NCR202819).

## Results

Of the total sample (N=1977), 1408 responses were excluded (327 due to ineligibility and 1081 due to suspicious responses) and 569 were retained. Most surveys eliminated were dated October 9-11, 2020 (n=1072). These dates align with the period when the survey link was posted on social media.

Comparisons of retained and excluded respondents showed statistically significant differences across most demographic characteristics (Table 2). There were lower rates of cisgender male, transgender/gender fluid/two-spirit identification (*P*<.001) and higher rates of cisgender female identification (*P*<.001) among retained versus excluded respondents. There was a higher prevalence of straight-identifying respondents in the retained sample versus the excluded sample (*P*<.001). There were lower rates of respondents reporting Native American/Alaska Native/Pacific Islander race/ethnicity (*P*<.001) and higher rates of those reporting White race/ethnicity in the retained sample versus the excluded sample (*P*<.001). The numbers of single individuals were similar in the two samples, but higher rates of divorced/separated and widowed people were observed in the retained sample versus the excluded sample (*P*<.001). There were higher rates of college completion and graduate school as well as annual incomes greater than US $100,000 among the retained sample versus the excluded sample (*P*<.001). The mean age of the retained sample was significantly older (56 vs 42 years old, *P*<.001).

**Table 2:** Differences between respondents in the retained and excluded samples.

Characteristic		Retained sample (n=569)	Excluded sample (n=1081)	*P* value	
Current age (years), mean (SD)		55.9 (13.1)	41.4 (8.2)	<.001	
**Gender, n (%)^a,b^**					
	Cisgender male	132 (23.2)	575 (53.2)	<.001	
	Transgender male, transgender female, gender fluid, or two-spirit	1 (0.2)	32 (3.0)	<.001	
	Cisgender female	399 (70.1)	463 (42.8)	<.001	
	Other/prefer not to answer/Do not understand the question	40 (7.0)	14 (1.3)	<.001	
**Sexual orientation, n (%)^b^**					<.001
	Straight	532 (93.5)	984 (91.0)		
	Lesbian, gay, homosexual, bisexual/pansexual, queer, two-spirit	23 (4.0)	89 (8.2)		
	Other/prefer not to answer/do not understand the question	14 (2.5)	8 (0.7)		
**Race/ethnicity, n (%)^a,b^**					
	Asian	19 (3.3)	58 (5.4)	.06	
	Black	83 (14.6)	200 (18.5)	.045	
	Hispanic/Latinx	42 (7.4)	90 (8.3)	.50	
	Native American/Alaska Native/Pacific Islander	17 (3.0)	83 (7.7)	<.001	
	White	411 (72.2)	677 (62.6)	<.001	
**Partnership status, n (%)^b^**					<.001
	Single	93 (16.3)	152 (14.1)		
	Married/partnered	388 (68.2)	884 (81.8)		
	Divorced/separated	60 (10.5)	37 (3.4)		
	Widowed	28 (4.9)	8 (0.7)		
Number of individuals in household, mean (SD)		2.6 (1.3)	3.3 (0.9)	<.001	
**Education, n (%)^b^**					<.001
	Some high school or less	17 (3.0)	38 (3.5)		
	High school diploma or GED^c^/vocational school	88 (15.8)	294 (27.2)		
	Some college	164 (28.8)	415 (38.4)		
	Completed 4-year degree	156 (27.4)	261 (24.1)		
	Graduate school	144 (25.3)	73 (6.8)		
**Annual household income (US $), n (%)^b^**					<.001
	<25,000	59 (10.4)	46 (4.3)		
	25,001-50,000	106 (18.6)	383 (35.4)		
	50,001-75,000	124 (21.7)	375 (34.7)		
	75,001-100,000	61 (10.7)	182 (16.8)		
	>100,000	129 (22.7)	93 (8.6)		
	I prefer not to answer	90 (15.8)	1 (0.09)		
Age at cancer diagnosis (years), mean (SD)		51.4 (13.4)	36.8 (8.6)	<.001	
**Cancer stage, n (%)^b^**					<.001
	I	172 (30.2)	456 (42.2)		
	II	167 (29.4)	367 (34.0)		
	III	88 (15.5)	177 (16.4)		
	IV	62 (10.9)	51 (4.7)		
	Unknown	66 (11.6)	24 (2.2)		
**Cancer type, n (%)^a,b^**					
	Melanoma	26 (4.6)	57 (5.3)	.53	
	Lung	23 (4)	199 (18.4)	<.001	
	Prostate	37 (6.5)	90 (8.3)	.19	
	Breast	328 (57.6)	161 (14.9)	<.001	
	Colorectal	39 (6.9)	117 (10.8)	.008	
	Kidney	8 (1.4)	63 (5.8)	<.001	
	Bladder	8 (1.4)	83 (7.7)	<.001	
	Blood cancer (leukemia, lymphoma, myeloma)	44 (7.7)	82 (7.6)	.92	
	Uterine/cervical	32 (5.6)	160 (14.8)	<.001	
	Thyroid	31 (5.5)	91 (8.4)	.03	
	Other	62 (10.9)	13 (1.2)	<.001	
**Time since cancer treatment (years), n (%)^b^**					<.001
	<2	238 (43.4)	476 (44.1)		
	2-5	168 (30.7)	488 (45.2)		
	>5	142 (25.9)	116 (10.7)		
**Cancer care status, n (%)^a,b^**					
	My cancer is in remission or no evidence of disease	447 (78.6)	612 (56.6)	<.001	
	I have chronic cancer	77 (13.5)	240 (22.2)	<.001	
	I am receiving palliative care	30 (5.3)	253 (23.4)	<.001	
	I am in hospice care	0 (0)	60 (5.6)	<.001	
	None of these apply to me	42 (7.4)	39 (3.6)	<.001	
Part of a tribe or territory, n (%)^b^		41 (7.2)	397 (38.1)	<.001	
**Employment status, n (%)^a,b^**					
	Retired	198 (34.8)	48 (4.4)	<.001	
	Paid work (full- or part-time)	251 (44.1)	667 (61.7)	<.001	
	Unpaid work (homemaker, volunteer)	44 (7.7)	127 (11.8)	.01	
	Unemployed	77 (13.5)	247 (22.9)	<.001	
**Insurance type, n (%)^a,b^**					
	Private insurance	320 (56.2)	436 (40.3)	<.001	
	Medicaid	83 (14.6)	491 (45.4)	<.001	
	Medicare	210 (36.9)	633 (58.6)	<.001	
	Tricare/COBRA^d^/other	48 (8.4)	64 (5.9)	.054	
	I do not have health insurance	31 (5.5)	45 (4.2)	.24	
**Self-reported health, n (%)^b^**					<.001
	Excellent/very good	165 (29.0)	375 (34.7)		
	Good	226 (39.7)	318 (29.4)		
	Fair	101 (17.8)	254 (23.5)		
	Poor	17 (3.0)	133 (12.3)		

^a^Respondents could select multiple responses for this question.

^b^Responses may not add up to n=569 or n=1081 due to missing data or multiple responses.

^c^GED: General Educational Development.

^d^COBRA: Consolidated Omnibus Budget Reconciliation Act.

The samples also differed in cancer stage, type, health status, and insurance coverage status. The retained sample reported more stage IV cancer and a higher percentage of breast cancer than the excluded sample. The excluded sample reported more lung, kidney, bladder, and uterine/cervical cancers than the retained sample (*P*<.001). A greater percentage of the retained versus excluded sample reported completing treatment more than 5 years ago (142/569, 25.9%, vs 116/1081, 10.7%; *P*<.001). A greater percentage of those in the retained sample indicated their cancer was in remission or had no evidence of disease (447/569, 78.6%, vs 612/1081, 56.6%; *P*<.001), while a greater percentage of the excluded sample reported receiving palliative care (253/1081, 23.4%, vs 30/569, 5.3%; *P*<.001) and hospice (60/1081, 5.6%, vs 0/569, 0%; *P*<.001). A greater percentage of the retained sample reported having private insurance (320/569, 56.2%), while more of the excluded sample reported having Medicaid (491/1081, 45.4%) and/or Medicare (633/1081, 58.6%). Finally, respondents in the retained sample were more likely to report their health as “good” (226/569, 39.7%, vs 318/1081, 29.4%) and less likely to report their health as “poor” (17/569, 3.0%, vs 133/1081, 12.3%) compared to the excluded sample (*P*<.001).

## Discussion

### Principal Findings

Numerous indications support the greater integrity of the data in the retained sample (n=569) compared to the excluded sample (n=1081). First, discordant data reported by the same respondent, such as the anatomical site of their cancer not being physically possible for their reported sex/gender, were clear signs of random survey completion. Second, the younger mean age of the excluded sample combined with cancers more likely to be diagnosed at a later age (eg, lung, kidney, and bladder cancers), more serious disease (chronic, receiving palliative care, or hospice), and poorer health is highly suspicious. Conversely, the higher self-reported diagnosis of breast cancer in the retained sample aligns with the authors’ prior research experience in more easily recruiting breast cancer survivors than those with a history of other cancers.

This study contributes to the literature by providing guidance for identifying potentially fraudulent data. Importantly, use of screening questions and CAPTCHA was insufficient to dissuade fraudulent respondents. Consistent with past research, we found that examining repeated personal data across responses [11], duplicate open text responses [12], response inconsistency [12], and low-probability responses [12] helped to identify potentially fraudulent responses. Additionally, we found that examining differences between the retained and excluded samples bolstered our confidence in the retained sample (ie, demographic characteristics such as mean age and cancer type corresponded more closely with the demographics of participants in prior cancer survivorship research conducted by the authors as well as cancer statistics).

### Ethical Considerations

Social media is an efficient and cost-effective method for health research. However, the potential for loss of data integrity must be weighed with the efficiency and cost-effectiveness [1-5]. The distance created between researchers and participants in internet survey–based research may lead to participants feeling less self-conscious about unethical behavior and more motivated to obtain incentives for which they are ineligible. Precautions to improve confidence in data integrity, however, may inadvertently prevent participation by eligible persons as well. For example, persons using the same computer who are eligible to participate in a study may be omitted from data based on their identical IP addresses. People with less technological savvy or visual challenges may be dissuaded from survey completion by the CAPTCHA. People whose first language does not match the language of the survey may be dissuaded due to instructions to type words in a language in which they are not facile. Finally, the capture of geographic location (IP address) in combination with multiple identifying questions has implications for the anonymity of respondents. Prevention and detection of fraudulent responses may, therefore, require increased justification for IRB review to collect geolocation and identifying data that would not otherwise be needed.

### Recommendations to Prevent Fraudulent Data

To minimize bot contamination and reduce duplicate entries, precautions similar to those taken in this study are warranted. Additional recommendations include using software with fraud prevention and detection capabilities (eg, Qualtrics), capturing IP addresses, capturing time stamps for both start and stop times, including a required open text question, and distributing surveys only to closed groups on social media or avoiding social media altogether. If social media is used, financial incentives should be avoided. If providing financial incentives, (1) require participants to check a box indicating they acknowledge that responses from ineligible respondents or those who respond multiple times will not receive the financial incentive and downplay the incentive, and (2) indicate that investigators reserve the right to confirm eligibility by telephone (or other means) and include a required telephone number field.

### Recommendations to Identify Fraudulent Data

Once data are collected, data integrity checks such as those in Table 2 can help researchers detect potentially fraudulent responses. In addition, the use of different trackable URLs for different dissemination channels may facilitate the identification of the dissemination source of suspicious data.

### Limitations

The criteria used to eliminate responses were subjective, and it is impossible to know if all fraudulent data were removed. The authors erred on the side of potentially eliminating valid responses rather than retaining responses that were likely to be invalid. Limitations in our ability to detect potentially fraudulent responses included the inability to capture IP addresses or completion times.

### Conclusion

Providing a survey incentive in combination with social media recruitment may increase the likelihood of fraudulent activity. CAPTCHA alone is unlikely to prevent fraudulent responses in internet-based research promoted on social media. Precautions to prevent and detect fraud are important for the validity of research findings. Ethical considerations of participant privacy and incentive payments should be weighed with data integrity concerns to ensure valid, meaningful health research results.

## Abbreviations

CAPTCHA: Completely Automated Public Turing test to tell Computers and Humans Apart
COC: Commission on Cancer
IRB: Institutional Review Board
REDCap: Research Electronic Data Capture
